# Duration of Carbapenemase-Producing *Enterobacteriaceae* Carriage in Hospital Patients

**DOI:** 10.3201/eid2609.190592

**Published:** 2020-09

**Authors:** Yin Mo, Anastasia Hernandez-Koutoucheva, Patrick Musicha, Denis Bertrand, David Lye, Oon Tek Ng, Shannon N. Fenlon, Swaine L. Chen, Moi Lin Ling, Wen Ying Tang, Timothy Barkham, Niranjan Nagarajan, Ben S. Cooper, Kalisvar Marimuthu

**Affiliations:** National University Hospital, Singapore (Y. Mo);; Mahidol-Oxford Tropical Medicine Research Unit, Bangkok, Thailand (Y. Mo, A. Hernandez-Koutoucheva, P. Musicha, B.S. Cooper);; National University of Singapore, Singapore (Y. Mo, D. Lye, S.L. Chen, N. Nagarajan, K. Marimuthu);; Genome Institute of Singapore, Singapore (D. Bertrand, S.N. Fenlon, S.L. Chen, N. Nagarajan);; University of Oxford, Oxford, UK (P. Musicha, B.S. Cooper);; Nanyang Technological University (D. Lye, O.T. Ng);; National Centre for Infectious Diseases, Singapore (D. Lye, O.T. Ng, K. Marimuthu);; Tan Tock Seng Hospital, Singapore (D. Lye, W.Y. Tang, T. Barkham, K. Marimuthu);; Singapore General Hospital, Singapore (M.L. Ling)

**Keywords:** Carbapenemase-producing Enterobacteriaceae, antimicrobial resistance, infection prevention and control, carriage, bacteria

## Abstract

To determine the duration of carbapenemase-producing *Enterobacteriaceae* (CPE) carriage, we studied 21 CPE carriers for »1 year. Mean carriage duration was 86 days; probability of decolonization in 1 year was 98.5%, suggesting that CPE-carriers’ status can be reviewed yearly. Prolonged carriage was associated with use of antimicrobial drugs.

Rapid global dissemination of carbapenemase-producing *Enterobacteriaceae* (CPE) poses a public health threat (*1*). To prevent the spread of CPE in healthcare settings, international guidelines advocate for early identification, isolation, and contact precautions (*2*,*3*). To provide information helpful for the design of rational infection control policies, we estimated CPE carriage duration in a hospital cohort and identified risk factors for prolonged carriage.

## The Study

During October 2016–February 2018, we conducted a prospective cohort study involving CPE carriers from 2 tertiary care centers in Singapore. CPE carriers were identified by routine collection of rectal swab samples in accordance with local infection control policies. We included patients who were >21 years of age and had the capacity to provide consent (Appendix Figure 1). We retrieved from medical records of enrolled patients the latest dates of CPE-negative rectal swab samples before the first positive sample. We collected fecal samples from participants at the time of enrollment, weekly for 4 weeks, monthly for 5 months, and bimonthly for 6 months. We recorded demographic characteristics, healthcare contact history, and medication history.

The fecal samples were inoculated onto selective chromogenic agar (CHROMID CARBA SMART; bioMérieux, https://www.biomerieux-diagnostics.com), and species identification was performed with matrix-assisted laser desorption/ionization time-of-flight mass spectrometry (Bruker, https://www.bruker.com). Antimicrobial susceptibility testing was performed by using VITEK-2 (bioMérieux). All *Enterobacteriaceae* isolates with a MIC of >2 mg/L for meropenem or >1.0 mg/L for ertapenem underwent PCR to detect *bla*_NDM-1_, *bla*_KPC_, *bla*_OXA-48_, *bla*_IMI-1_, and *bla*_IMP_ genes (*4*). All CPE isolates and fecal DNA underwent sequencing on an Illumina HiSeq 4000 sequencer (https://www.illumina.com). We used the Shannon diversity index to measure α-diversity for fecal microbial communities (https://cran.r-project.org/web/packages/vegan/vegan.pdf).

We analyzed data by using Bayesian multistate Markov models to account for interval censoring (Appendix). First, we estimated the overall transmission rates by considering patients to be in either CPE colonized or noncolonized states. Second, we considered CPE colonization on the species level and included as separate states carbapenemase-producing (CP)–*Escherichia coli* (CP-EC) colonized, CP–*Klebsiella pneumoniae* (CP-KP) colonized*,* and CP-EC/KP co-colonized (Appendix Figure 2). All analyses were performed by using R version 3.4.4 (https://www.R-project.org) and RStan (http://mc-stan.org).

We enrolled 21 patients (Table). Mean (± SD) follow-up period was 294 (± 77) days, and each participant provided 12 (± 1.5) samples. Throughout follow-up, 15 (71.4%) participants carried >1 species of CPE, and only 3 (14.3%) carried >1 type of carbapenemase gene (χ^2^ of difference in proportions = 14.8, simulated p = 0.0005) (Appendix Table 1). The most common species carried were *K. pneumonia*e (18 [85.7%]) and *E. coli* (16 [76.2%]). The most frequently observed carbapenemase genes were *bla*_OXA-48_ (11 [52.4%]) and *bla*_KPC_ (8 [38.1%]). We obtained 76 CP-KP isolates from the samples; the most common sequence type (25 [32.9%]) was 307. Among the 83 CP-EC isolates, the most common sequence type (22 [26.5%]) was 131. Sample positivity was continuous until clearance for most (17 [81.0%]) of the 21 participants. For 4 participants, negative samples were followed by positive samples; the longest period was 3 negative samples over 3 consecutive weeks (Appendix Figure 5).

**Table:** Demographics for 21 participants in study of duration of carbapenemase-producing *Enterobacteriaceae* carriage in hospital patients, Singapore*

Characteristic	No. (%)
Sex	
M	15 (71.4)
F	6 (28.6)
Ethnicity	
Chinese	15 (71.4)
Malay	3 (14.3)
Indian	2 (9.5)
Other	1 (4.8)
Ambulatory status	
Independently performs ADL	12 (57.1)
Requires assistance in ADL	4 (19.0)
Wheelchair bound	3 (14.3)
Bed bound	2 (9.5)
Recent surgery†	15 (71.4)
Colonization or infection with another MDRO in the year preceding enrollment	4 (19.0)
Hospitalization in past year	11 (52.4)
Antibiotic intake during follow-up period	13 (61.9)
Readmission during follow-up period	10 (47.6)
Recent overseas travel	13 (61.9)

*Median age (interquartile range) was 60.0 (50.0–69.0) y; median Charlson Morbidity Index (interquartile range) 3.0 (2.0–5.0). ADL, activities of daily living; MDRO, multidrug-resistant organisms, including methicillin-resistant *Staphylococcus aureus*, vancomycin-resistant *Enterococcus*, carbapenem-resistant *Enterobacteriaceae*, carbapenem-resistant *Acinetobacter baumanii*, and carbapenem-resistant *Pseudomonas aeruginosa*, recorded from surveillance and clinical cultures taken in the year before study enrollment. †Gastrointestinal surgeries (n = 7), skin and soft tissue surgeries (n = 4), neurosurgery (n = 2), removal of Tenckhoff catheter (n = 1), and urologic procedure (n = 1).

The estimated mean duration of CPE carriage was 86 (95% credible interval [CrI] 60– 122) days. The probability of decolonization in 1 year was 98.5% (95% CrI 95.0%–99.8%), assuming a constant decolonization rate within the time interval. The longest observed carriage duration was 387 days. We performed a sensitivity analysis that included 16 participants who became decolonized during follow-up (i.e., the last sample collected was negative for CPE). This analysis gave a mean carriage time of 77 (95% CrI 53–108) days and a 98.8% (95% CrI 96.5%–99.9%) probability of decolonization within 1 year.

As time-fixed covariates, we analyzed age, co-colonization with other multidrug-resistant organisms, presence of a urinary catheter, antimicrobial drug use during follow-up, Charlson Comorbidity Index score, and readmission; as a time-varying covariate, we used the Shannon Diversity Index score to explore the covariates’ association with decolonization (Appendix Figure 3). The only factor associated with prolonged CPE carriage was antimicrobial drug use during the follow-up period (hazard ratio 0.48, 95% CrI 0.20–0.93). The rate of decolonization for CP-EC was lower than that for CP-KP (0.018 [95% CrI 0.007–0.031] per day vs. 0.030 [95% CrI 0.016–0.049] per day) (Appendix Table 2, Figure 4).

## Conclusions

CPE infections are typically preceded by asymptomatic carriage, especially in vulnerable patients such as those who are immunocompromised and critically ill (*5*). To prevent transmission, active surveillance to identify CPE carriers is essential but may be associated with a high cost:benefit ratio if implemented without knowledge of the natural history of CPE carriage.

Previously reported CPE carriage durations vary widely; median durations range from 43 to 387 days (*5**–**7*). These variations probably result from differences in follow-up schedules, microbiological and molecular methods used to identify CPE, and criteria to define clearance. Studies that reported longer carriage duration tended to adopt an opportunistic sampling strategy and considered both clinical and fecal samples to determine carriage (*7*). Opportunistic sampling may lead to selection bias because patients with more healthcare contacts would have more samples collected. Infrequent and inconsistent sampling is more likely to misclassify recolonization from a new transmission event as continuous colonization, resulting in perceived longer duration of carriage.

Of note, the participants carried more species of CPE than types of carbapenemase genes. Although this observation may be the result of new acquisition events, it is more parsimoniously explained by active interspecies horizontal gene transfer, especially in a low-transmission setting such as Singapore. Differential rates of clearance of CP-KP and CP-EC can be related to colonizing affinity of the species and fitness cost of carbapenemase genes, which vary widely among different species (*8*). Further studies incorporating between-host and within-host transmission dynamics of resistance may shed light on the roles of bacterial clones and plasmids in spreading and maintaining resistance.

Our study has limitations. First, because the participants were screened after hospital admission, the time of initial colonization could not be confidently determined. However, our multistate Markov models assume a constant rate of decolonization. Our sensitivity analysis used the latest CPE-negative swab samples, so carriage duration estimates were similar to those calculated without the last known CPE-negative swab samples, suggesting that our modeling assumptions were reasonable. Second, our sample size was small and drawn from a single population, and the extent to which our findings can be extrapolated to other populations is uncertain. However, our study was rigorously conducted in terms of frequency of fecal sample collection, duration of follow-up, and number of participants in a nonoutbreak setting. The use of multistate models has been shown to preserve power with modest sample size given more frequent follow-ups (*9*).

Our systematic sampling and robust methods for identifying CPE enabled us to closely follow participants’ carriage status. Using combined detection methods of culture on carbapenem-resistance selective media, antibiotic susceptibility testing, and PCR, we found that 4 (19.0%) patients had intervening negative samples, 1–3 weeks apart. This finding suggests that a patient should have >2 negative samples 4–6 weeks apart before considering CPE to be eliminated. The finding that the probability of decolonization is 98.5% in 1 year suggests that a policy of reviewing CPE carrier status yearly may be appropriate for this population. Further health economics analysis is needed to make institution-specific recommendations for rescreening frequency. Given the finding that antimicrobial drug use was the most important factor associated with prolonged CPE carriage, use of antimicrobial drugs in these patients should be avoided if not clinically indicated.

Appendix Supplementary methods and results for study of duration of carbapenemase-producing *Enterobacteriaceae* carriage in hospital patients.
