# Supplementary material for: Duration of Carbapenemase-Producing Enterobacteriaceae Carriage in Hospital Patients
Source: Emerg Infect Dis. 2020 Sep;26(9):2182–5. doi: 10.3201/eid2609.190592 (PMC7454053; doi:10.3201/eid2609.190592)
Supplement: Appendix — Supplementary methods and results for study of duration of carbapenemase-producing Enterobacteriaceae carriage in hospital patients. [file 19-0592-Techapp-s1.pdf]

Article DOI: https://doi.org/10.3201/eid2609.190592

Duration of Carbapenemase-Producing *Enterobacteriaceae* Carriage in Hospital Patients

Appendix

Supplemental Material 1: Methodology

Microbiology

Microbiological cultures to identify CPE were performed via direct inoculation of stool samples onto selective and indicative agar, chromID® CARBA SMART Agar (CARB/OXA, Biomerieux). After overnight incubation, colonies were identified at the species level with matrix assisted laser desorption ionization-time of flight-mass spectrometry (MALDI-ToF-MS, Bruker Daltonics GmHB, Bremen, Germany). Phenotypic antimicrobial susceptibility testing was performed using VITEK-2 (bioMérieux Vitek, Inc., Hazelwood, MO). All *Enterobacteriaceae* isolates with a MIC to meropenem ≥2mg/L, or Ertapenem MIC ≥1.0mg/L, underwent polymerase chain reaction (PCR) to test for the presence of blaNDM-1, blaKPC (blaKPC-2 to blaKPC-13), blaOXA48, blaIMI-1 and blaIMP carbapenemase genes, as previously reported. (Teo *et al*, Singapore J Med Microbiol. 2013)

Genomic analysis

Library preparation for DNA from CPE isolates was performed using the NEBNext® Ultra DNA Library Prep Kit for Illumina®. Sequencing with 2x151bp reads was performed using the Illumina HiSeq 4000 sequencer.

Raw FASTQ reads were processed using standard in-house pipelines. Briefly, MLST and antibiotic resistance genes were called directly from raw reads as well as from de novo assemblies; discrepancies between these were resolved with manual examination of both types of data. For analysis of raw reads, MLST and antibiotic resistance genes were called directly from the FASTQ files using SRST2 (version 0.1.8) (Inouye *et al*, Genome Med. 2014) with default settings using the ARGAnnot database provided with the SRST2 distribution for resistance

genes. De novo assemblies were performed using the Velvet assembler (version 1.2.10) (Zerbino *et al*, Genome Res. 2008) with parameters optimized by the Velvet Optimiser script packaged with the velvet distribution, scaffolded with Opera (version 1.4.1) (Gao *et al*, J Comput Biol. 2011), and finished with FinIS (version 0.3) (Gao *et al*, Algorithms in Bioinformatics 2012). Genomes were annotated with Prokka (version 1.11) (Seemann *et al*, Bioinformatics. 2014). For analysis of de novo assemblies, resistance genes were called using BLASTN with a minimum identity of 90% over 90% of the gene length required for calling a gene present, using the same ARGAnnot database as used by SRST2. MLST calls were made by using a custom BLASTN-based MLST caller. The MLST databases were downloaded using the SRST2 helper scripts from https://pubmlst.org.

Shotgun metagenomics

DNA from stool samples was extracted using PowerSoil® DNA Isolation Kit (12888, MOBIO Laboratories) with modifications to the manufacturer's protocol. To avoid spin filter clogging, we extended the centrifugation to twice the original duration and solutions C2, C3 and C4 were doubled in volume. DNA was eluted in 80µL of Solution C6. Concentration of DNA was determined by Qubit dsDNA BR assay (Q32853, Thermo Fisher Scientific). For the library construction, 50ng of DNA was re-suspended in a total volume of 50µL and was sheared using Adaptive Focused Acoustics (Covaris) with the following parameters; Duty Factor: 30%, Peak Incident Power (PIP): 450, 200 cycles per burst, Treatment Time: 240s. Sheared DNA was cleaned up with 1.5× Agencourt AMPure XP beads (A63882, Beckman Coulter). Gene Read DNA Library I Core Kit (180434, Qiagen) was used for end-repair, A-addition and adaptor ligation. Custom barcode adapters (HPLC purified, double stranded, 1st strand: 5′ P-GATCGGAAGAGCACACGTCT; 2nd strand: 5′ ACACTCTTTCCCTACACGACGCTCTTCCGATCT) were used in replacement of Gene Read Adaptor I Set for library preparation. Library was cleaned up twice using 1.5× Agencourt AMPure XP beads (A63882, Beckman Coulter). Enrichment was carried out with indexed-primers according to an adapted protocol from Multiplexing Sample Preparation Oligonucleotide kit (Illumina). We polled the enriched libraries in equi-molarity and sequenced them on an Illumina HiSeq sequencing instrument to generate 2 × 101 bp reads, yielding 17,744 million paired-end reads in total and 49 million paired-end reads on average per library.

Reads were processed with an in-house shotgun metagenomics data analysis pipeline (https://github.com/CSB5/shotgun-metagenomics-pipeline). Read quality trimming was performed using famas (https://github.com/andreas-wilm/famas, v0.10,–no-order-check), and bacterial reads were identified by mapping to the human reference genome hg19 using bwa-mem (v0.7.9a, default parameters).

Microbial community taxonomic profiles were obtained using MetaPhlAn (v2.0, default parameters, relative abundance >0.01%) which provides relative abundances of bacteria, fungi and viruses at different taxonomic levels. The Shannon diversity index was computed from species-level taxonomic profiles using the function diversity from the R library vegan. The detection of antibiotic resistance genes was performed using SRST2 (v0.1.4, fraction of gene covered >99%) using the predefined ARGAnnot database. (Appendix Table 1, Figures 1,2)

To estimate rates of colonization and duration of carriage, we modeled colonization and carriage dynamics using multi-state Markov models. First, we considered CPE to be a homogeneous bacterial group and at any sampling time point patients belonged to one of two states: "non-colonised" or "colonised ". Secondly, we considered CPE by species with patients being "not colonized by any CPE," "colonized by CP-*E. coli*," "colonized by CP-*K. pneumoniae*" or colonised by "both CP-*E. coli* and *K. pneumoniae*," resulting in a four-state Markov model. In either model, transitions from one state to another was governed by a $K \times K$ intensity matrix Q (where *K* is the number of states). For $r \neq s$, the rate of transition from state r to state s, $q_{rs} = Q[r, s]$ was modeled by the linear equation:

$$\log\left(q_{rs}\right) = \beta_{0+} \sum_{i=1}^{D} \beta_i \times X_i$$

where $\{X_1, X_2, \ldots, X_D\}$ is a set of covariates.

For the two-state homogeneous CPE model we included age, taking antibiotics during follow-up, Charlson comorbidity index, co-colonization with other multidrug resistant organisms, infection with CPE, readmission, and presence of urinary catheter as time-fixed covariates, and Shannon diversity index as a time-varying covariate.

We implemented the models in STAN modeling language within the R environment. We used N~(0,1) as prior distributions for parameters estimates, while posterior distributions were

sampled using the Hamiltonian Markov Chain Monte Carlo method. The prior is a generic weakly informative prior as recommended by Rstan which contains enough information to regularize i.e., rule out unreasonable values but is not so narrow as to rule out probable values (Gelman, Github. 2019, https://github.com/stan-dev/stan/wiki/Prior-Choice-Recommendations). Posterior distributions were sampled from 4 chains run over 20,000 iterations (including 10,000 burn-in) and we assessed model convergence using the Gelman-Rubin convergence diagnostic statistic and through visualization of trace plots. (Appendix Table 2).

Appendix Table 1. Carbapenamase producing *Enterobacteriaceae* and associated plasmids carried by 21 participants throughout the study period

CPE species	No. (%)
Klebsiella pneumoniae (%)	18 (85.7)
Eschericia coli (%)	16 (76.2)
Enterobacter spp (%)	5 (23.8)
Citrobacter spp (%)	3 (14.3)
Co-colonization by >1 CPE species during follow up	15 (71.4)
Types of carbapenem-resistance genes	
OXA-48 (%)	11 (52.4)
KPC (%)	8 (38.1)
NDM-1 (%)	3 (14.3)
IMP (%)	2 (9.5)
Co-colonization by >1 type of plasmid during follow up	3 (14.3)

*Percentages refer to proportion of participants carrying the bacterial species or carbapenem-resistance genes. A single participant may carry more than one type of bacterial species or carbapenem-resistance genes during the observational period.

Appendix Table 2. Rates of the four-state multistate model with CP-*E. coli*, CP-*Klebsiella*, carrying both CP-*E. coli* and CP-*Klebsiella* (Both), CPE-non-carrying state (None)

State transitions	Event /day	95% CrI
CP-*E. coli* to none	0.018	0.007 – 0.031
CP-*Klebsiella* to none	0.030	0.016 – 0.050
CP-*E. coli* to both	0.041	0.018 – 0.078
CP-*Klebsiella* to both	0.045	0.019 – 0.087
Both to CP-*E. coli*	0.037	0.014 – 0.076
Both to CP-*Klebsiella*	0.040	0.018 – 0.073

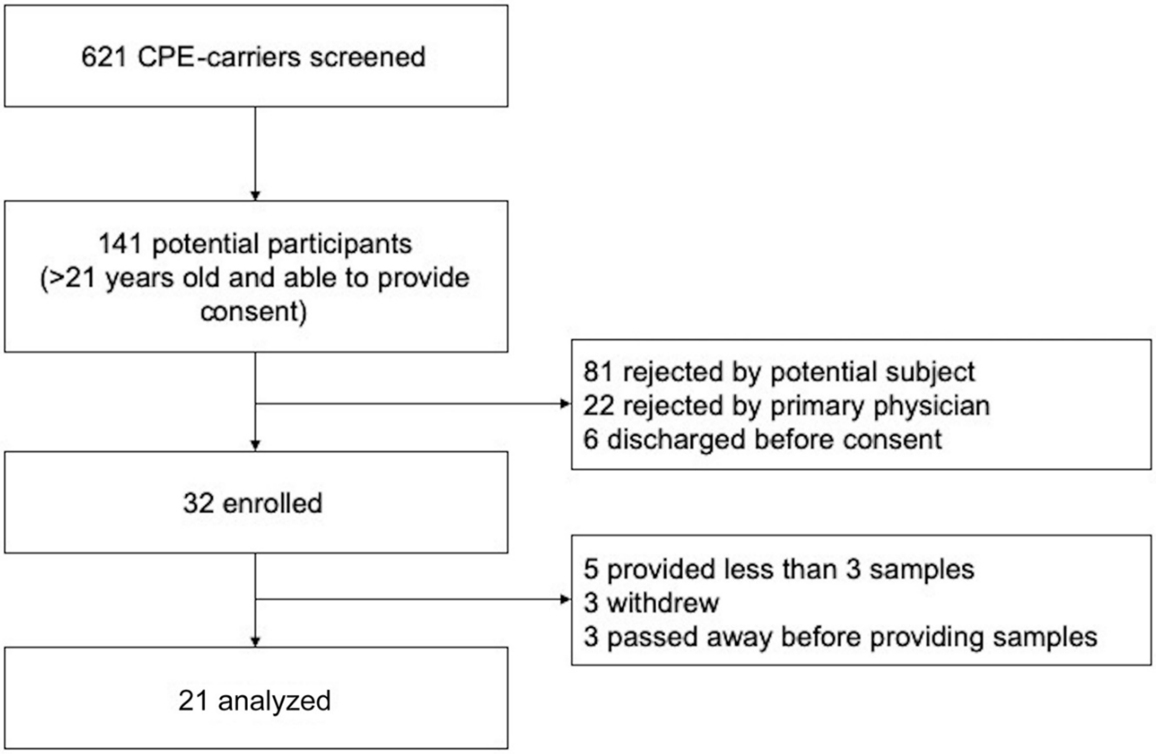

Appendix Figure 1. Recruitment flow diagram.

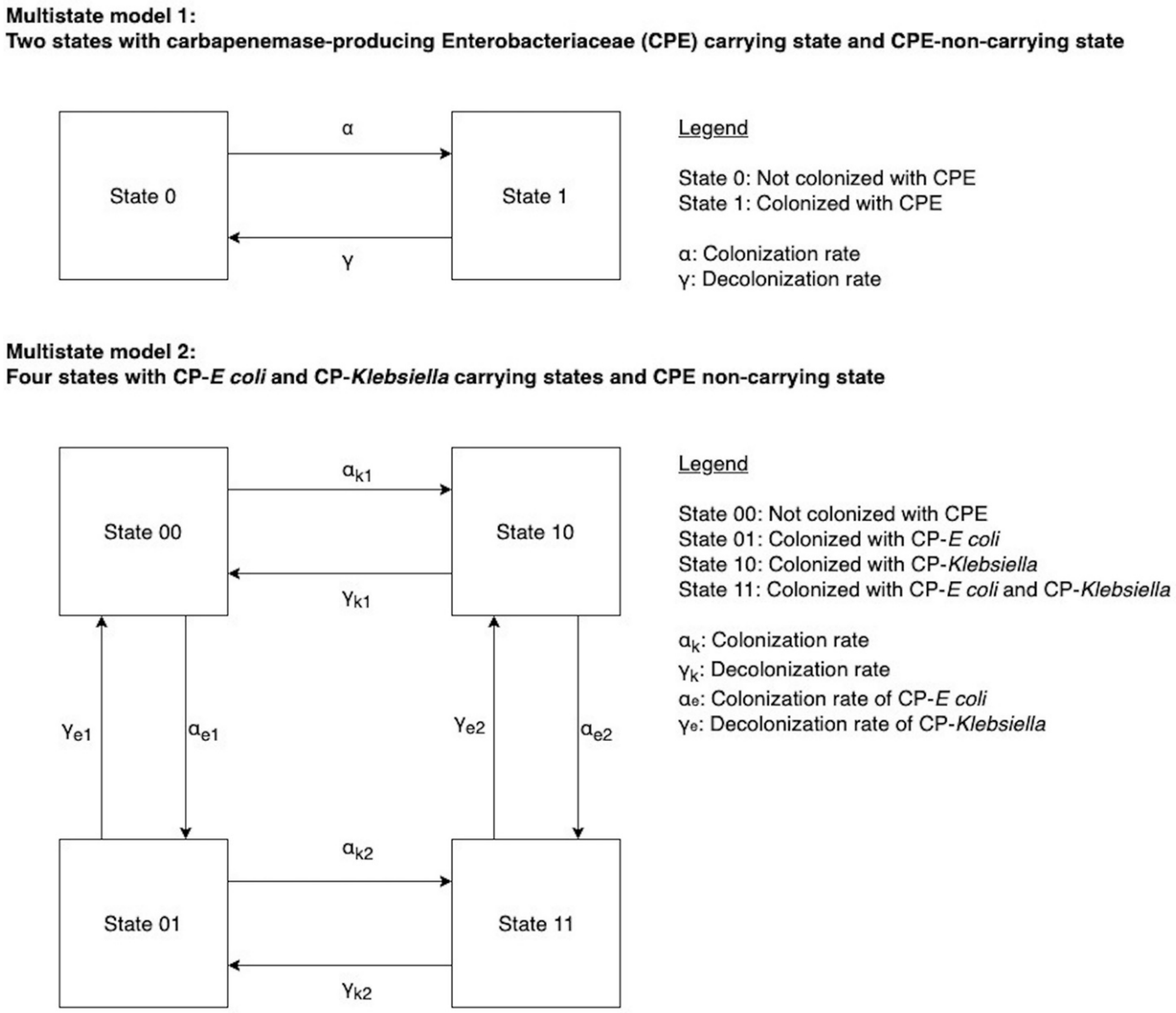

Appendix Figure 2. Multistate Markov models for the analysis of CPE carriage

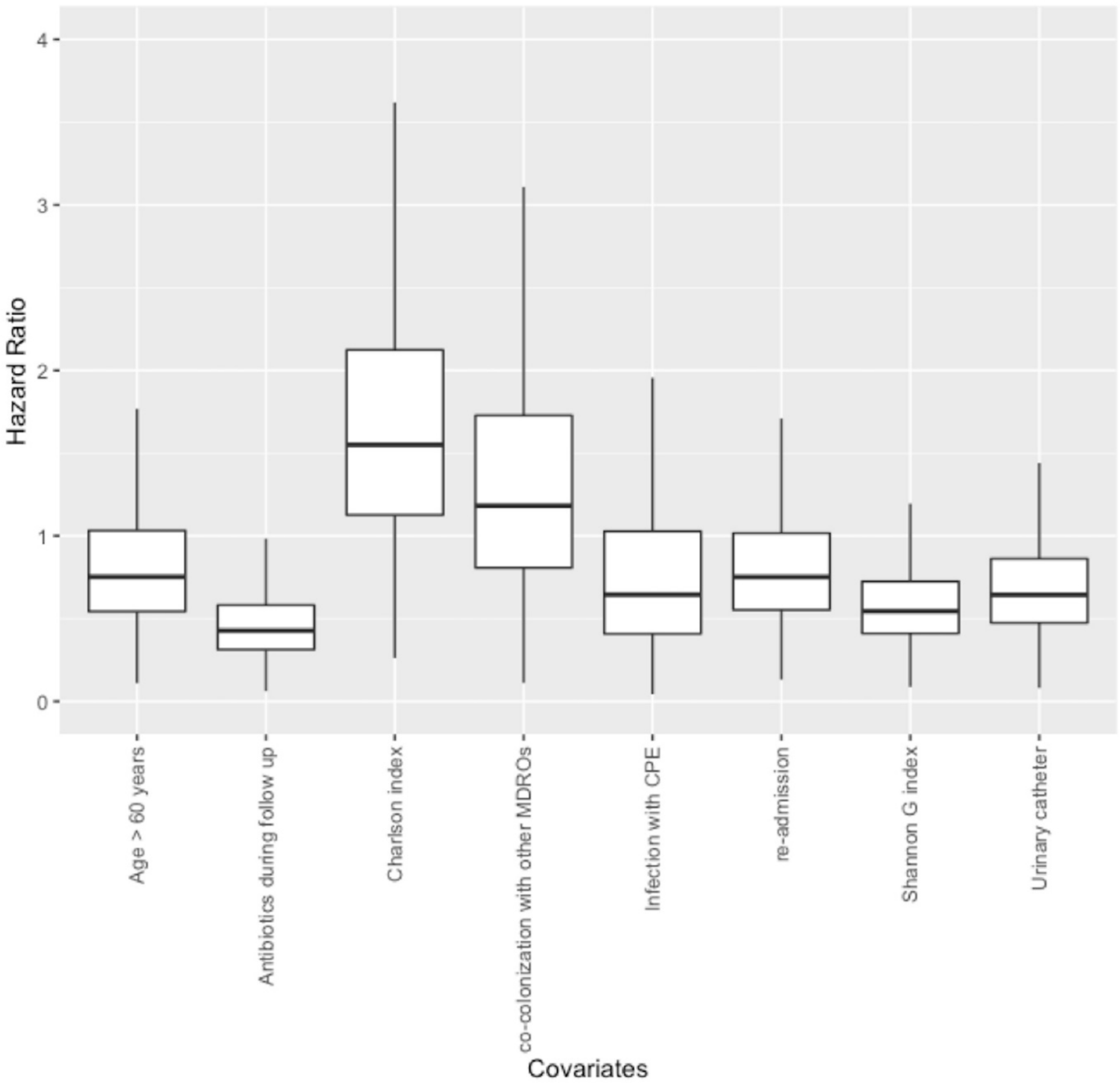

Appendix Figure 3. Associations of potential risk factors of CPE colonization and decolonization rate.

MDRO: Multidrug-resistant organisms including methicillin-resistant *Staphylococcus aureus*, vancomycin-resistant *Enterococcus*, carbapenem-resistant *Enterobacteriaceae*, carbapenem-resistant *Acinetobacter baumanii* and carbapenem-resistant *Pseudomonas aeruginosa*.

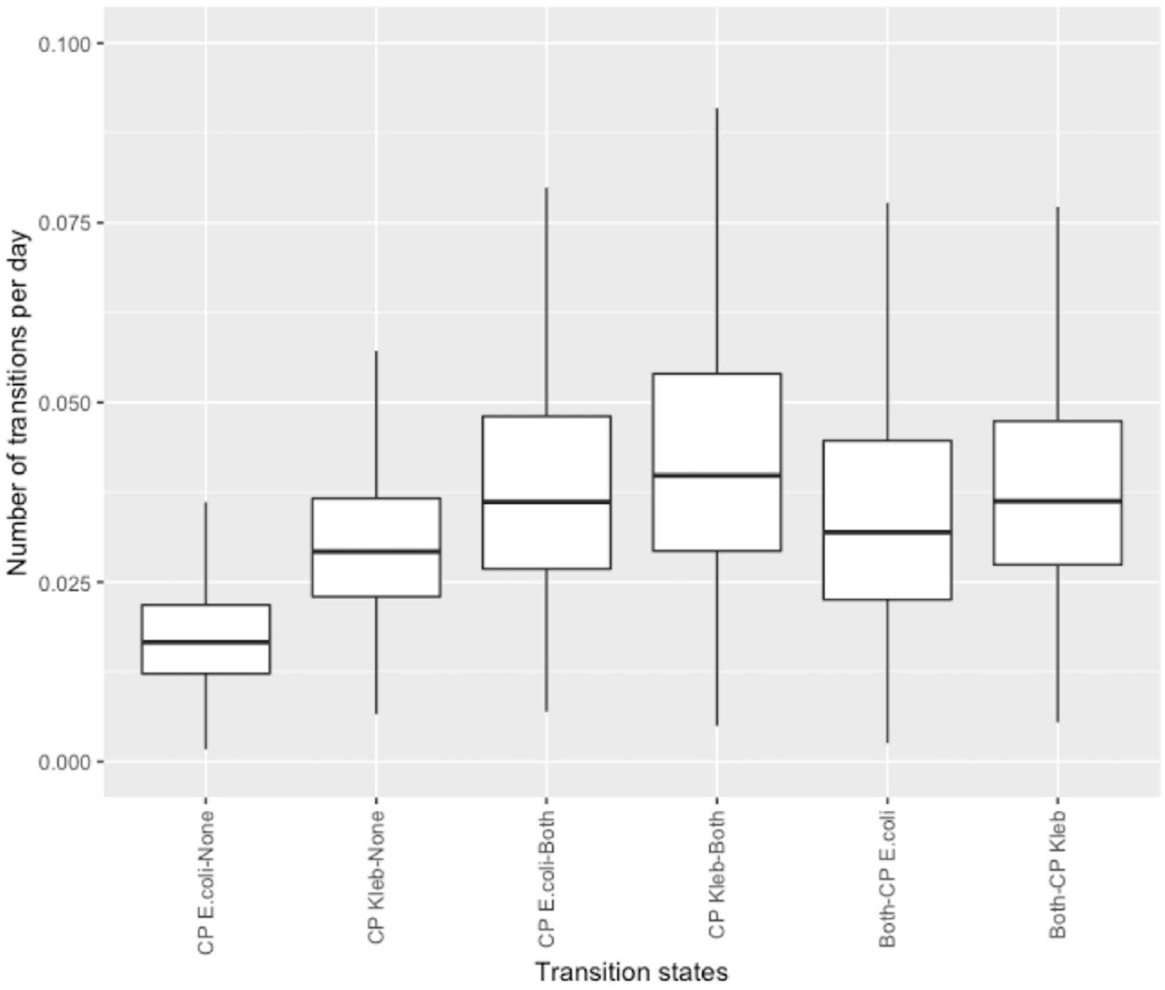

Appendix Figure 4. Rates of the four-state multistate model where CP-*E. coli and* CP-*Klebsiella pneumoniae* are separate states.

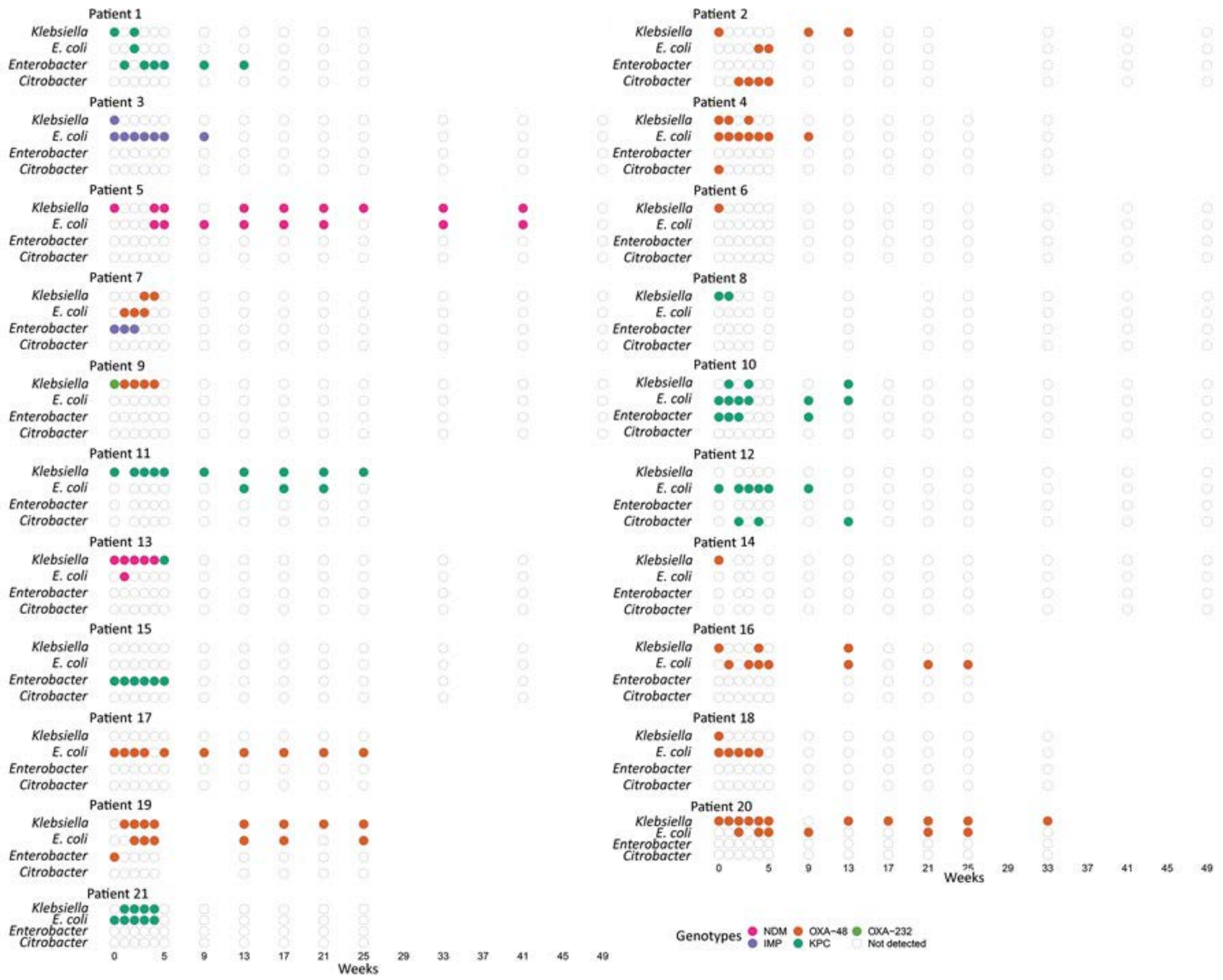

Appendix Figure 5. Types of carbapenemase-resistant *Enterobacteriaceae* and plasmid colonization for participants in study of duration of carbapenemase-producing *Enterobacteriaceae* carriage in hospital patients, Singapore. Microbiological outcomes of fecal samples from each participant are shown. Each column of dots represents 1 fecal sample. Each dot represents an *Enterobacteriaceae* species, including *Klebsiella spp., Escherichia coli, Enterobacter spp.*, *and Citrobacter spp.*, from the fecal samples carrying carbapenemase-resistant *Enterobacteriaceae* genes. Missing data are shown by the absence of dots on a particular week.
